# Revisión narrativa de estudios por imágenes de las calcificaciones de la glándula submandibular

**DOI:** 10.21142/2523-2754-1101-2023-143

**Published:** 2023-03-26

**Authors:** Marco Rozas Pozo, Gustavo Adolfo Fiori-Chíncaro, Jhoana Mercedes Llaguno-Rubio

**Affiliations:** 1 Division de Radiologia Bucal y Maxilofacial, Instituto Latinoamericano de Altos Estudios en Estomatologia. Lima, Peru. mrozaspozo@outlook.com, gfiori@ilaeperu.com, academico@ilaeperu.com Division de Radiologia Bucal y Maxilofacial Instituto Latinoamericano de Altos Estudios en Estomatologia Lima Peru mrozaspozo@outlook.com gfiori@ilaeperu.com academico@ilaeperu.com

**Keywords:** sialolitiasis, glándula submandibular, sialolito, sialolithiasis, submandibular gland, sialolith

## Abstract

La sialolitiasis es una de las patologías más comunes de las glándulas salivales mayores y se presenta con mayor frecuencia en las glándulas submandibulares. Entre el 80 y el 95% de los sialolitos se desarrollan en las glándulas submandibulares; entre el 5 y el 20%, en la glándula parótida; y solo el 1%, en la glándula sublingual. Los sialolitos se forman dentro del parénquima y los sistemas de conductos asociados. En el conducto de Wharton, del 80 al 90%, y solo el 15% en la glándula. La sialolitiasis es la causa del dolor e inflamación de la glándula salival al obstruir el conducto y evitar la secreción salival, antes, durante y después de la ingestión de los alimentos. El objetivo de esta revisión fue realizar la descripción actualizada de los distintos métodos de diagnóstico por imágenes, utilizados para el estudio de las calcificaciones de la glándula submandibular, a partir de diferentes estudios reportados en la literatura científica contemporánea, con la finalidad de establecer el diagnóstico correcto. Se realizó una búsqueda de la literatura en las principales fuentes de información, incluidas Medline (vía PubMed), Elsevier, SciELO y LILACS, usando los términos de búsqueda con una limitación de fecha de los últimos 5 años en promedio. Los artículos seleccionados incluyeron información referente a calcificaciones de las glándulas salivales. Los estudios por imágenes de las calcificaciones de las glándulas salivales se pueden obtener con radiografías convencionales, sialografía, ultrasonografía (US), tomografía computarizada (TC) y resonancia magnética (RM).

## Abstract (en)

Sialolithiasis is one of the most common pathologies of the major salivary glands and occurs more frequently in the submandibular glands. Between 80 and 95% of sialoliths develop in the submandibular glands, between 5 and 20% in the parotid gland, and only 1% in the sublingual gland. Sialoliths form within the parenchyma and associated duct systems. In Wharton's duct (80-90%) and only 15% in the gland. Sialolithiasis is the cause of pain and inflammation of the salivary gland by obstructing the duct and preventing salivary secretion, before, during and after food.

The objective of this article was to review the different diagnostic imaging methods used for the study of calcifications of the submandibular gland, based on different studies reported in contemporary scientific literature, in order to establish the correct diagnosis. A search of the literature was carried out in the main information sources including Medline (via PubMed), SEVIER, SCIELO, and LILACS, using the search terms with a date limitation of the last 5 years on average. The selected articles included information regarding the calcifications of the salivary glands. Imaging studies of salivary gland calcifications can be obtained with conventional radiographs, Sialography, Ultrasonography (US), Computed Tomography (CT) and Magnetic Resonance Imaging (MR).

## INTRODUCCIÓN

La sialolitiasis es una de las patologías más comunes de las glándulas salivales mayores, después de la parotiditis y la más común de las glándulas submandibulares ^1^^,^^2^. Los sialolitos se forman dentro del parénquima y los sistemas de conductos asociados ^3^^,^^4^. En el conducto de Wharton, del 80 al 90%, y solo el 15% en la glándula ^5^^,^^6^. Entre el 80 y el 95% de los sialolitos se desarrollan en las glándulas submandibulares; entre el 5 y el 20%, en la glándula parótida; y solo el 1%, en la glándula sublingual ^7^. Es más común en adultos jóvenes; 12 de cada 1000 sufren sialolitiasis y los varones son los más afectados ^1^^,^^7^. Los niños se afectan muy raramente ^3^^,^^8^^,^^9^.

La sialolitiasis afecta por igual a las glándulas de ambos lados y los casos bilaterales son menos frecuentes ^6^. En cuanto al tamaño, el 88% tiene menos de 10 mm y los que tienen un tamaño superior a 15 mm se consideran gigantes, pero son muy raros ^10^^,^^11^. La sialolitiasis es la causa del dolor y la inflamación de la glándula salival, ya que obstruye el conducto y evita la secreción salival antes, durante y después de ingerir los alimentos ^10^^,^^12^.

Los estudios por imágenes de las calcificaciones de las glándulas salivales se pueden obtener con radiografías convencionales, sialografía, ultrasonografía (US), tomografía computarizada (TC) y resonancia magnética (RM) ^4^^,^^9^^,^^13^^-^^15^.

Las calcificaciones de tejido blando en el área maxilofacial son de frecuencia variable y pueden encontrarse en el 4% en las radiografías panorámicas ^16^; generalmente, corresponden a hallazgos radiográficos en exámenes de rutina. Los criterios diagnósticos son la ubicación anatómica, la distribución, el número, la forma y el tamaño de las calcificaciones ^17^.

El diagnóstico diferencial incluye a flebolitos, tonsilolitos, ateromas de la arteria carótida y nodos linfáticos submandibulares calcificados ^18^^-^^22^. En los últimos años, se ha incrementado el uso de la tomografía computarizada de haz cónico (TCHC) en la práctica odontológica ^23^^,^^24^, lo que tiene algunas ventajas y está indicado en el caso de sialolitiasis compleja ^25^^-^^29^. Sin embargo, la TCHC no debe considerarse la primera opción para el diagnóstico y tratamiento de sialolitiasis ^23^, pues resulta necesario conocer las otras alternativas para lograr un buen diagnóstico. Por esto, el propósito de este estudio fue realizar la revisión de la información científica contemporánea existente sobre las calcificaciones de la glándula submandibular y la utilización de los métodos de diagnóstico por imágenes, con la finalidad de contribuir a un mejor proceso de diagnóstico. 

## MATERIALES Y MÉTODOS

Se realizó una búsqueda de la literatura en las principales fuentes de información en buscadores como Medline (vía PubMed), Elsevier, SciELO y LILACS, usando las palabras clave “glándula submandibular”, “calcificaciones de las glándulas salivales”, “sialolitos” y “sialolitiasis”. También se buscaron artículos relacionados con el tema en revistas especializadas del área como *Oral Surg Oral Med Oral Pathol Oral Radiol, Dentistry Journal, Journal of Oral Science, Imaging Science in Dentistry, Científica dental, European Radiology, Avances en Odontoestomatología, Revista Odontológica Mexicana, Indian J Dent Res, J Cranio-Maxillofacial Surg, Int J Oral Maxillofac Surg* y *Dentomaxillofacial Radiol*.

Se incluyeron estudios descritpivos, observacionales, retrospectivos y series de casos; así mismo, se excluyeron revisiones sitemáticas, monografias, artículos de opinión, editoriales y cartas al editor. La información incluida correspondió a los últimos 5 años, con el fin de presentar la información más actualizada sobre el tema de estudio. Finalmente, se revisó 55 artículos que fueron analizados en los distintos tópicos descritos.

## RESULTADOS Y DISCUSIÓN

### Histología y anatomía de la glándula submandibular

Las glándulas salivales mayores constituyen estructuras pares, bilaterales. Incluyen las glándulas parótida, submandibular y sublingual, cuya función es secretar y excretar saliva hacia la cavidad bucal ^30^. Histológicamente, comprenden múltiples unidades secretoras que, básicamente, son conglomerados de células serosas y mucosas ^28^^,^^31^. La sialona es la unidad glandular estructural y funcional de las glándulas salivales, que consta de una pieza secretora o acino, un conducto intercalar, un conducto estriado y un conducto excretor ^29^^,^^32^.

Las glándulas salivales están conformadas por parénquima y estroma. El parénquima contiene los acinos y los conductos de secreción. El estroma se compone de tejido conectivo que le da sostén y células mioepiteliales con capacidad contráctil, ubicadas entre la membrana basal y las células salivales, lo que facilita la secreción del contenido salival de las glándulas ^28^^,^^29^. Las glándulas parótidas contienen principalmente acinos serosos; en cambio, las glándulas sublinguales están formadas por acinos mucosos y las glándulas submandibulares son mixtas, formadas por acinos serosos y mucosos ^29^^,^^32^.

### Etiología y epidemiologia de las calcificaciones de la glándula submandibular

Los factores etiopatogénicos asociados a la sialolitiasis están caracterizados por la obstrucción mecánica y la disminución del flujo salival, debido a la formación de sialolitos en el parénquima o conducto excretor de la glandula ^33^^-^^35^. La obstrucción puede ser completa o parcial y recurrente. 

Los cálculos de las glándulas salivales se forman como resultado de la deposición de sales de calcio alrededor de un núcleo o matriz orgánica, formado por descamación de células epiteliales, bacterias, moco y cuerpos extraños. Su estructura es laminar y tienen como principal componente inorgánico a los fosfatos y pequeñas cantidades de carbonatos de calcio en forma de hidroxiapatita, con cantidades pequeñas de potasio, magnesio y amoniaco ^1^^,^^36^. La producción de saliva viscosa, mucosa y alcalina de la glándula submandibular, relativamente alta en hidroxiapatita y carbonato apatita, predispone a la precipitación de sales; además, la apertura del conducto de Wharton es más estrecha que el diámetro del conducto completo y es ascendente desde la glándula hasta su apertura en el piso de la boca, lo que favorece la retención de saliva y la formación de las calcificaciones ^13^^,^^37^.

Los sialolitos son más comunes en personas entre la cuarta y sexta década de la vida, y es más frecuente en varones ^34^. Son raros en la población pediátrica ^6^^,^^7^. El 80% de los sialolitos se presentan en la glándula submandibular; el 6%, en la glándula parótida; y el 2%, en la glándula sublingual. Se presentan, usualmente, de manera unilateral y tienen formas redondas u ovoides, de superficie áspera o lisa, y color amarillento ^38^.

Los factores etiopatogénicos que tradicionalmente se asocian a las calcificaciones en las glándulas salivales son la obstrucción, la disminución del flujo salival, la deshidratación, las modificaciones del pH de la saliva, que está relacionado con una sepsis orofaríngea y dificultades en la solubilidad de los cristaloides ^30^.

Los procesos inflamatorios de las glándulas salivales mayores pueden ser agudas o crónicas, y están relacionadas, aunque no siempre, a la enfermedad obstructiva, especialmente en la glándula submandibular, donde la obstrucción genera una sialoadenitis, que es una inflamación que afecta principalmente al parénquima acinar de la glándula. Las infecciones se deben a diferentes microorganismos, como bacterias aerobias y anaerobias, virus, hongos y micobacterias (flora bacteriana mixta). Los microorganismos que habitualmente se encuentran son el *Staphylococcus aureus* y el *Streptococcus* spp. El flujo lento y disminuido de la saliva condiciona el flujo retrógrado del *Staphylococcus aureus* y especies de *Streptococcus* hacia el interior de los conductos y de la propia glándula, lo cual produce la infección ^30^^,^^39^.

En el diagnóstico clínico diferencial de la glándula submandibular, se consideran la ránula, el mucocele, los abscesos sublinguales y otras alteraciones inflamatorias e infecciosas del piso de la boca. Otras patologías semejantes son asintomáticas, como las linfadenopatías ^34^.

En cuanto al diagnóstico imagenológico diferencial, se considera las imágenes radiopacas intraóseas sobreproyectadas: la enostosis, la exostosis, la osteoesclerosis y la osteítis condensante ^18^^,^^34^.

### Estudios por imágenes

#### a. Radiografías panorámicas y oclusales

Las radiografías panorámicas y oclusales son parte esencial de los diagnósticos estándar en la mayoría de la práctica odontológica. Las radiografías bidimensionales se han utilizado para diagnosticar sialolitos submandibulares y las calcificaciones de las glándulas salivales se presentan como hallazgos en radiografías de rutina ^39^^,^^40^.

En un estudio comparativo para evaluar el rendimiento diagnóstico de las radiografías panorámicas y oclusales en la detección de sialolitos mandibulares, Kim *et al*. ^41^^)^ concluyeron que las técnicas radiográficas, tanto panorámicas como oclusales, mostraron un rendimiento diagnóstico satisfactorio y deben considerarse antes de utilizar una tomografia computarizada para detectar sialolitos submandibulares, a menos que la determinación de la ubicación tridimensional de un sialolito sea necesaria para la planificación quirúrgica. Sin embargo, en ocasiones, debemos modificar la técnica oclusal para visualizar un cálculo salival que, por su ubicación, no podemos detectar.

Aproximadamente, el 20% de los sialolitos están poco calcificados y no son visibles en las radiografías bidimensionales ^41^^-^^43^. La modificación de los factores eléctricos juega un papel importante para la detección de un sialolito radiotransparente; por consiguiente, se debe ajustar la dosis de radiación para evitar la sobreexposición de los sialolitos ^44^^,^^45^.

#### b. Sialografía

La sialografía consiste en la opacificación de los conductos salivales mediante la inyección intracanalicular retrógrada del medio de contraste. Los medios de contraste utilizados para los estudios sialográficos son las soluciones acuosas o hidrosolubles (sinografin), y las oleosas o liposolubles (ethiodol y lipiodol); estos medios de contraste contienen concentraciones relativamente elevadas (25-40%) de yodo. Los medios acuosos tienen más facilidad para mezclarse con las secreciones salivales y mayor facilidad de distribución en los conductos más delgados. Los medios oleosos son más viscosos y requieren una presión de inyección mayor que los medios acuosos para visualizar los conductos más finos ^30^^,^^39^.

Las desventajas de la sialografía incluyen la dosis de irradiación, el dolor relacionado con el procedimiento, la posibilidad de perforación de la pared del canal y las complicaciones de la infección; además, este procedimiento tiende a empujar el sialolito más al fondo del canal y es contraindicada en los procesos agudos y la reacción alérgica al medio de contraste ^36^^,^^43^^,^^46^.

Un sialograma completo tiene tres fases bien diferenciadas de acuerdo con el momento en el que se toman las radiografías después de la inyección del material de contraste. La fase ductal permite la observación de los conductos mayores; la fase acinar muestra el sistema ductal opacificado por completo, lo cual produce el relleno posterior del parénquima de la glándula con el medio de contraste; y la fase de evacuación valora la función secretora normal de la glándula para determinar si hay residuos del material de contraste en la glándula o en el sistema de conductos después del sialograma, durante más de cinco minutos después de haberse inyectado la sustancia de contraste ^30^.

#### c. Tomografía computarizada (TC)

Es utilizada generalmente para la valoración de las lesiones en masa de las glándulas salivales (tumores benignos y malignos), pero además puede demostrar la presencia de cálculos en las glándulas submandibulares, localizados en la parte posterior del conducto y en el hilio de la glándula. Asimismo, es menos invasiva que una sialografía y no necesita material de contraste ^30^.

Los sialolitos pueden ser radiopacos o radiotransparentes, pero estos últimos son difíciles de diagnosticar con las técnicas radiográficas convencionales. Actualmente, las técnicas tridimensionales, como la tomografía computarizada multicorte (TCMC) y la tomografía computarizada de haz cónico (TCHC), permiten una delimitación mucho mejor del cálculo y el sistema de conductos de las glándulas salivales ^21^^,^^30^.

Existe la posibilidad de encontrar dificultades en el diagnóstico de la sialolitiasis por síntomas similares a patologías dentales y no dentales en el área maxilofacial. En una serie de casos, Kolomiiets *et al*. ^47^ informaron tres casos de sialolitiasis en la glándula salival submandibular que mostraban similitudes sintomáticas con otros trastornos dentales y no dentales, y cuyos diagnósticos fueron confirmados por estudios imagenológicos tridimensionales con tomografía computarizada (3D-CT). El primer caso correspondía a una paciente con pericoronitis del tercer molar mandibular derecho parcialmente impactado, con diagnóstico final de sialolitiasis de glándula submandibular, por un sialolito radiotransparente. El segundo caso fue un ameloblastoma hallado radiográficamente y que hizo que la sialolitiasis fuera imperceptible por al menos nueve años, a pesar de la radiopacidad del sialolito. El tercer caso estuvo relacionado con un osteoma detectado radiográficamente y problemas de endodoncia con el segundo molar mandibular izquierdo, que fueron los hallazgos clínicos iniciales preferenciales. Un examen clínico e imagenológico con 3D-CT confirmo un diagnóstico de sialolitiasis submandibular.

Cuando no se ha llegado al diagnóstico a través de otros métodos y el cuadro clínico persiste, la tomografía helicoidal multicorte es una buena alternativa, pues aprovecha las técnicas de reconstrucción multiplanar y en tercera dimensión, además de la navegación virtual por los conductos salivales. La TCMC puede mostrar con éxito, incluso, los cálculos más pequeños o semicalcificados, aunque a expensas de las altas dosis de radiación para los pacientes ^15^^,^^48^.

#### d. Tomografía computarizada de haz cónico (TCHC)

La disponibilidad, el bajo costo y las dosis más bajas de radiación de la TCHC, en comparación con las imágenes de la TCMC, son un aporte valioso que la convierten en una buena alternativa para el diagnóstico imagenológico no invasivo de la sialolitiasis ^26^. Así, “Los sistemas de tomografía computarizada de haz cónico (TCHC) se han diseñado para obtener imágenes de los tejidos duros de la región maxilofacial. TCHC es capaz de proporcionar una resolución submilimétrica en imágenes de alta calidad de diagnóstico, con tiempos de exploración cortos (10 a 70 segundos) y dosis de radiación, según se informa, hasta 15 veces más bajas que las de las exploraciones por TC convencionales” ^24^.

Los cálculos salivales se pueden evaluar adecuadamente con TCHC, mientras que las mediciones de las calcificaciones son altamente reproducibles y difieren muy poco de las mediciones realizadas con US. La sensibilidad y la especificidad con TCHC son más altas que las obtenidas con otros métodos de diagnóstico por imágenes. Debido a su alta relación entre la información diagnóstica y la dosis de radiación, la TCHC es la modalidad de imagen de elección para el diagnóstico de calcificaciones en las glándulas salivales ^15^^,^^25^.

En una investigación realizada por You Meng *et al*. ^49^, utilizando TCHC, se estudió a 84 pacientes con sialolitos radiopacos submandibulares y se detectó un total de 128 sialolitos en 84 personas, de las cuales 22 (26,19%) tenían sialolitos múltiples. Clasificaron los sialolitos en cinco tipos morfológicos: manchas, redondas, esferoides, alargadas e irregulares, con la forma esferoide como la más frecuente. Se correlacionó el tamaño y la ubicación de los sialolitos, con el sialolito grande que se ubicó en la parte posterior del conducto. El 39,06% (50/128) de los sialolitos se ubicaban en la parte anterior del conducto y el 60,94% (78/128) se localizó en la parte posterior. Concluyendo que las imágenes de TCHC mostraron datos importantes para la evaluación, el diagnóstico y el plan de tratamiento de la sialolitiasis submandibular. 

Asimismo, se pueden realizar procedimientos de sialografía combinados con TCHC, con la finalidad de lograr mejores resultados para la visualización de los conductos, como el realizado por Drage y Brown ^40^ en dos pacientes con obstrucción de la glándula submandibular. La sialografía con TCHC puede mejorar la sensibilidad, la especificidad y la correlación espacial de los hallazgos en los conductos de las glándulas salivales ^43^.

#### e. Ultrasonografía (US)

Es un método de imagen inocuo, no invasivo, de bajo costo, ampliamente disponible y fácil de realizar por su corto tiempo de exploración; además, permite una valoración exacta de las glándulas salivales ^30^^,^^50^. La US tiene la capacidad de clasificar las lesiones intra y extraglandulares, así como de caracterizar la presencia de anomalías, y es particularmente útil para evaluar la sospecha de sialolitiasis o procesos agudos submandibulares. El espacio submandibular y las estructuras que contiene son superficiales y de fácil acceso para el examen de US de alta resolución. La glándula submandibular es el componente principal del espacio submandibular ^5^.

La US muestra a la glándula submandibular como una estructura encapsulada y de ecotextura homogénea; los conductos intraglandulares se observan como estructuras lineales, hiperecoicas; el conducto de Wharton se ve mejor cuando está dilatado patológicamente y en sujetos normales se observa como una estructura tubular de paredes delgadas. Los cálculos se muestran como estructuras enérgicamente calcificadas en el interior de la propia glándula y son focos hiperecoicos con sombra acústica distal. Se ha informado que la US tiene una precisión de hasta el 96% para la detección de cálculos y es la elección inicial para la investigación de sospecha de sialolitiasis. Las piedras impactadas en el conducto del ostium en el piso de la boca pueden no verse bien ecográficamente ^5^^,^^50^.

En un estudio realizado por Goncalves *et al*. ^51^ se informa sobre la valoración de la efectividad de la ecografía en el diagnóstico de sialolitiasis: “la sialolitiasis se puede diagnosticar mediante ecografía con un alto grado de certeza; por lo tanto, parece ser muy apropiada como método de elección para el examen”. Los sialolitos siempre se diagnosticaron en la ecografía en una ubicación intraparenquimatosa; sin embargo, señalan que los sialolitos no diagnosticados correctamente en la ecografía se ubicaron mayormente en el área distal del conducto, seguido por el tercio medio y, finalmente, en la parte proximal del conducto de Wharton.

#### f. Resonancia magnética (RM)

La RM es considerada superior a la TCMC en lo que se refiere a delimitar detalles de los tejidos blandos, en las lesiones de las glándulas salivales, sin exponer a los pacientes a radiaciones y sin la necesidad de utilizar medios de contraste para mejorar la imagen de las estructuras que se van a estudiar. Es una técnica nueva, no invasiva, que no causa molestias ni dolor asociado, y que consiste en realizar una reconstrucción volumétrica que permite la visualización de los conductos y su condición ^52^.

La reconstrucción tridimensional con RM, complementada con la endoscopía virtual del sistema de conductos, también por RM, arrojan buenos resultados para la visualización de las anomalías en la sialolitiasis y otros trastornos, como el síndrome de Sjögren, quistes, tumores y procesos inflamatorios ^30^^,^^52^.

Sus desventajas tienen que ver con los 45 minutos necesarios para la reconstrucción, el costo del equipo y del estudio, la intolerancia al examen por pacientes claustrofóbicos y las limitantes por la presencia de metales en la cavidad oral, como coronas de acero, cromo y amalgamas ^36^.

Sin embargo, la RM aún tiene limitaciones importantes. Si bien permite la evaluación del sistema ductal y muestra el edema glandular y el flemón, ha demostrado una capacidad limitada para demostrar calcificaciones en las glándulas salivales. Las imágenes de resonancia magnética son capaces de identificar conductos dilatados y un defecto de relleno, o una zona de transición dentro de un conducto dilatado, pero no pueden señalar la diferencia entre piedras, burbujas de aire, residuos inflamatorios, tejidos inflamados y una estenosis. Sin embargo, a menudo se requiere una radiografía simple o una TC para la confirmación de las calcificaciones ^53^.

La sialografía de resonancia magnética no requiere canular el conducto, ya que no hay necesidad de utilizar medios de contraste para mejorar la imagen de las estructuras que se van a estudiar. La sialografía de resonancia magnética hace posible visualizar pequeños sialolitos hasta los conductos de tercer nivel con sensibilidad y especificidad de alrededor del 90%. Sin embargo, debido a la complejidad del procedimiento y los costos asociados, la imagen de resonancia magnética, aún no ha ingresado en el trabajo clínico de rutina ^30^^,^^43^^,^^51^.

## CONCLUSIONES

Las calcificaciones de tejido blando en el área maxilofacial son de frecuencia variable, que pueden encontrarse en un 4% en las radiografías panorámicas. Los criterios diagnósticos son los siguientes: ubicación anatómica, distribución, número, forma y tamaño de las calcificaciones. 

El diagnóstico diferencial incluye a los flebolitos, tonsilolitos, ateromas de la arteria carótida y nodos linfáticos submandibulares calcificados, por lo que las radiografías panorámicas son las imágenes más usadas en odontología y deben ser parte de un protocolo de diagnóstico con respecto a las calcificaciones de los tejidos blandos.

## Fuente de financiamiento:

Autofinanciado
